# Focal Peak Activities in Spread of Interictal-Ictal Discharges in Epilepsy with Beamformer MEG: Evidence for an Epileptic Network?

**DOI:** 10.3389/fneur.2013.00056

**Published:** 2013-05-14

**Authors:** Douglas F. Rose, Hisako Fujiwara, Katherine Holland-Bouley, Hansel M. Greiner, Todd Arthur, Francesco T. Mangano

**Affiliations:** ^1^Division of Neurology, Department of Pediatrics, Cincinnati Children’s Hospital Medical CenterCincinnati, OH, USA; ^2^Division of Neurosurgery, Department of Neurosurgery, Cincinnati Children’s Hospital Medical CenterCincinnati, OH, USA

**Keywords:** magnetoencephalography, beamformer, children, adolescents, intracranial EEG, outcome, network, localization

## Abstract

Non-invasive studies to predict regions of seizure onset are important for planning intracranial grid locations for invasive cortical recordings prior to resective surgery for patients with medically intractable epilepsy. The neurosurgeon needs to know both the seizure onset zone (SOZ) and the region of immediate cortical spread to determine the epileptogenic zone to be resected. The immediate zone of spread may be immediately adjacent, on a nearby gyrus, in a different lobe, and sometimes even in the contralateral cerebral hemisphere. We reviewed consecutive simultaneous EEG/MEG recordings on 162 children with medically intractable epilepsy. We analyzed the MEG signals in the bandwidth 20–70 Hz with a beamformer algorithm, synthetic aperture magnetometry, at a 2.5 mm voxel spacing throughout the brain (virtual sensor locations, VSLs) with the kurtosis statistic (*g*_2_) to determine presence of excess kurtosis (γ_2_) consistent with intermittent increased high frequency spikiness of the background. The MEG time series was reconstructed (virtual sensor signals) at each of these VSLs. The VS signals were further examined with a relative peak amplitude spike detection algorithm. The time of VS spike detection was compared to the simultaneous EEG and MEG sensor signals for presence of conventional epileptiform spike morphology in the latter signals. The time of VS spike detection was compared across VSLs to determine earliest and last VSL to show a VS spike. Seven subjects showed delay in activation across VS locations detectable on visual examination. We compared the VS locations that showed earliest and later VS spikes with the locations on intracranial grid locations by electrocorticography (ECoG) that showed spikes and both onset and spread of seizures. We compared completeness of resection of VS locations to postoperative outcome. The VS locations for spike onset and spread were similar to locations for ictal onset and spread by ECoG.

## Introduction

Non-invasive studies to predict regions of seizure onset are important for the planning of intracranial grid locations for epilepsy. The surgeon needs to know both the seizure onset zone (SOZ) and the region of immediate cortical spread to determine the epileptogenic zone (EZ) to be resected (Engel, 1996; Wiebe et al., 2001; Luders et al., 2006). The immediate zone of spread may be immediately adjacent, on a nearby gyrus, in a different lobe, and sometimes even in the contralateral cerebral hemisphere. Among non-invasive recording modalities, magnetoencephalography (MEG) has some advantages because of its very good time resolution and good spatial resolution, as signals are not altered by the skull (Knowlton et al., 2009; Stefan et al., 2011). Prediction of intracerebral locations of sources for recorded extracranial MEG signals requires mathematical source localization algorithms. Multiple categories of algorithms have been developed including single and multiple equivalent current dipoles (ECD), dipole scans such as multiple signal classification (MUSIC) (Mosher, 1999), distributed dipoles (current density) such as minimum norm estimate (MNE) (Tanaka et al., 2010), standardized low resolution brain electromagnetic tomography (sLORETA) (Pascual-Marqui, 2002), vector and scalar beamformer (Robinson et al., 2004; Sekihara et al., 2005), and multiple other algorithms each with particular strengths. Source localization algorithms in the beamformer category are spatial filters and have very good signal to noise resolution. The beamformer algorithm, as a spatial filter, can “tune” the MEG sensor array to enhance the signal arising in a specific location while diminishing brain signals arising from other locations.

The tuning process has been referred to as creating a “virtual sensor” (VS) at the chosen spatial location (VS location) and the resulting spatially filtered signal is referred to as a VS signal. The tuning can be repeated iteratively for multiple locations intracranially on the same recording of the MEG signal, such that the whole brain can be scanned sequentially to evaluate source contributions to the overall signal that was recorded at the magnetometer sensors. We chose to evaluate the capability of a particular scalar beamformer algorithm, synthetic aperture magnetometry (SAM), to detect onset and spread of interictal spikes, interictal bursts of spikes, and ictal discharges (Robinson et al., 2004).

## Methods

### Subjects

We initially reviewed consecutive simultaneous EEG/MEG recordings between January 2006 and December 2008 for 162 children and adolescents with medically intractable epilepsy who were admitted for non-invasive Phase I presurgical evaluation. All subjects were recorded during spontaneous sleep after sleep deprivation, conscious sedation with chloral hydrate, or general anesthesia with dexmedetomidine (Table 1). This study was performed under Cincinnati Children’s Hospital Medical Center Internal Review Board Protocol 2008-0403 as a retrospective review.

**Table 1:** **Summary subjects findings, surgical treatment, outcome**.

Case	Age (Yr/Mo)	Presurgical etiology	Seizure type	EEG/MEG findings	VS spike locations	Intralobar timing (ms)	Intrahem timing (ms)	Interhem timing (ms)	Phase II ictal onset	Surgery	Outcome	Pathological report
1	16 Y 1 M	TS	CPS	Spikes	L FP; L Ant T; R F; L F; L Ant T	+; <10*; 63	+; <10*; 50	+; <20*; 40; 50	L FP; L Ant F; L T	L ant F; L T	Seizure free 2 Y	TS
2	7 Y 8 M	UNK	CPS	Spike burst	R Ins; R Mes Sup P; R Post Sup F; R Mes Sup P; R Post Sup F; R Ins	–	+; 80; 240; 260; 300	–	R Sup T; R Inf P; R Ant P (spread slow also on ECoG)	R temp; R parietal	Seizures; hemispherotomy; seizure free 2 Y	Diffuse cortical dysplasia
3	2 Y 5 M	L P O cortical dysplasia	CPS? tonic, atonic, myoclonic	Diffuse burst	L P-O; L Post F; R P; R Post F	–	+; 43	+; 43; 173	L P-O	L P; L O; L Post T	Seizure free 2 Y	Focal cortical dysplasia, Palmini Type IIa, IIb
4	15 Y 7 M	S/P R F tumor resection	CPS	Spikes, sharp waves	R F (posterior and mesial to resection);R P	+20	+; 100		R sup F; R mid F; R inf F; R mid T	R F	Seizures; vid/EEG bilateral spikes, some seizures R hemisphere	–
5a	8 Y 8 M preop	UNK	L facial twitching, myoclonic tonic	Spikes, sharp waves	R Ant P; R Post P; L P; L Post F; L Post F; L P	+10	+; 33	+; 62	R sup post F; R sup ant P	R F	Seizures	–
5b	Postop	UNK	Seizure onset changed side	Spikes seen on MEG only	L Post F; L P	–	+; <10	–	ND, family declined	ND	Seizures	ND
6	1 Y 10 M	Encephalitis	CPS	Spikes Diff burst	L P; R P	+; 13; 26	+; 39	+; 26	Nophase II, not a candidate	Callosotomy	Stopped drop attacks	–
7	17 Y 7 M	TS	Bilateral tonic “shudder” of arms	Interictal and ictal bursts	L F; R F	+	+	+	No phase II, chose not to have surgery	ND	Seizures continue	ND

*L, left; R, right; FP, frontopolar; F, frontal; T, temporal; P, parietal; O, occipital; Ins, insula; oper, operculum; ant, anterior; post, posterior; sup, superior; inf, inferior; mes, mesial; lat, lateral; intrahem, intrahemispheric; interhem, interhemispheric; VS, virtual sensor; CPS, complex partial seizure. S/P, status post; Y, years; M, months; +, present; –, absent; ms, milliseconds; diff, diffuse; UNK, unknown; TS, tuberous sclerosis; ND, not done*.

*A small amplitude deflection was seen shortly after VS spike at earliest VS location although the most prominent VS spike occurred at later timing.

### Electromagnetic recordings

All subjects had simultaneous 23-channel scalp EEG and 275-channel MEG recorded with a whole-head CTF 275-channel magnetometer (VSM MedTech Systems Inc., Coquitlam, BC, Canada). Fiducial markers were placed at the nasion and left and right preauricular points and placements were photographed digitally. The same fiducial locations were marked with MRI visible targets. The MRI scans were thin slice, 1 mm in the sagittal plane. Each subject had a series of simultaneous MEG/EEG recordings for 2 and 10 min durations for a minimum recording time of 40 min. All subjects’ recordings were digitized at 300 or 600 Hz for the 10-min recording and additionally at 4000 Hz for the 2-min recordings.

### Data analysis

We first visually reviewed each EEG/MEG recording in the bandwidth 1–70 Hz for ictal and interictal discharges, and removed any sections of the recordings with non-cerebral artifact, particularly sections containing muscle artifact. We further analyzed the remaining sections of MEG signals in the bandwidth 20–70 Hz with a beamformer algorithm, SAM, at a 2.5 mm voxel spacing throughout the brain (VS locations, VSLs) at approximately 300,000 locations in the three dimensional grid. We applied the kurtosis statistic (*g*_2_) to the signals at each VS location to determine presence of excess kurtosis (γ_2_) consistent with intermittent increased high frequency spikiness of the background (Ukai et al., 2004; Kirsch et al., 2006; Ishii et al., 2008; Prendergast et al., 2013). The kurtosis statistic is a measure of the peakedness and thickness of the tails of a unimodal distribution (DeCarlo, 1997). A positive kurtosis indicates more “outliers” in the tails of the distribution than expected. For this application, the “outliers” are intermittent additional high frequency activity within a constrained 20–70 Hz bandwidth compared to the background brain noise distribution in that bandwidth (Robinson et al., 2004). The VS locations were ordered based on highest to lowest excess kurtosis. The first 20 VS locations with highest excess kurtosis were chosen for further examination. A short note of explanation: Because the signal at each VS location is first evaluated for kurtosis separately from all others, a VS location with very infrequent additional high frequency activity may register a higher kurtosis than locations with more frequent, yet still intermittent, additional high frequency activity occurrences. Also, the VS location with the highest kurtosis may not have the most frequent VS spikes or the earliest occurring of the spike components. Therefore, once the VS locations demonstrating increased kurtosis are identified, each location must still be evaluated to assess the nature and frequency of occurrence of the increased high frequency activity. For the further analysis, the MEG time series (VS signal) was reconstructed for each of these VS locations. The continuous VS signals for each VS location were evaluated with a simple adaptive spike detection algorithm based on peak amplitude relative to prior signal amplitude. An additional note here: sometimes a VS location with increased kurtosis does not show a spike in the reconstructed signal. In that case the increased high frequency activity may be low amplitude, infrequent and occur over a longer time span than the short time span of a spike. Thus, although increased activity is present, because it is more spread out over time, the activity does not rise up above the background as a visible spike. The opposite effect can also occur if spikes occur very frequently, for instance 2/s throughout the recording, which has happened in some of our pediatric patients. Then the spikes are a common, consistent feature of the background, contribute to the central “peak” of the distribution, and do not increase the kurtosis at the tails. Also, since we measured the kurtosis in the bandwidth 20–70 Hz, spikes in the original MEG/EEG signal, whose spectral power was confined below 20 Hz (this can happen in very young children), would not be detected by this method. Thus the reconstructed VS signals must always be compared to the simultaneous original MEG/EEG signals. The EEG, MEG, and the VS signals were displayed together as channels, with the VS signal at each VS location displayed as a separate VS channel (Figure 3A shows simultaneous EEG and VS channels, but not the MEG sensor channels because of space limitations). The time of each VS spike detection in each VS location (channel) was compared to the simultaneous original signals recorded at the EEG electrodes and MEG sensors (EEG/MEG sensor signals) for the presence of conventional epileptiform spike morphology in the latter signals. Only the VS spikes that were associated with conventional epileptiform spikes in the EEG/MEG sensor signals were chosen for further analysis and evaluation.

**Figure 1:**
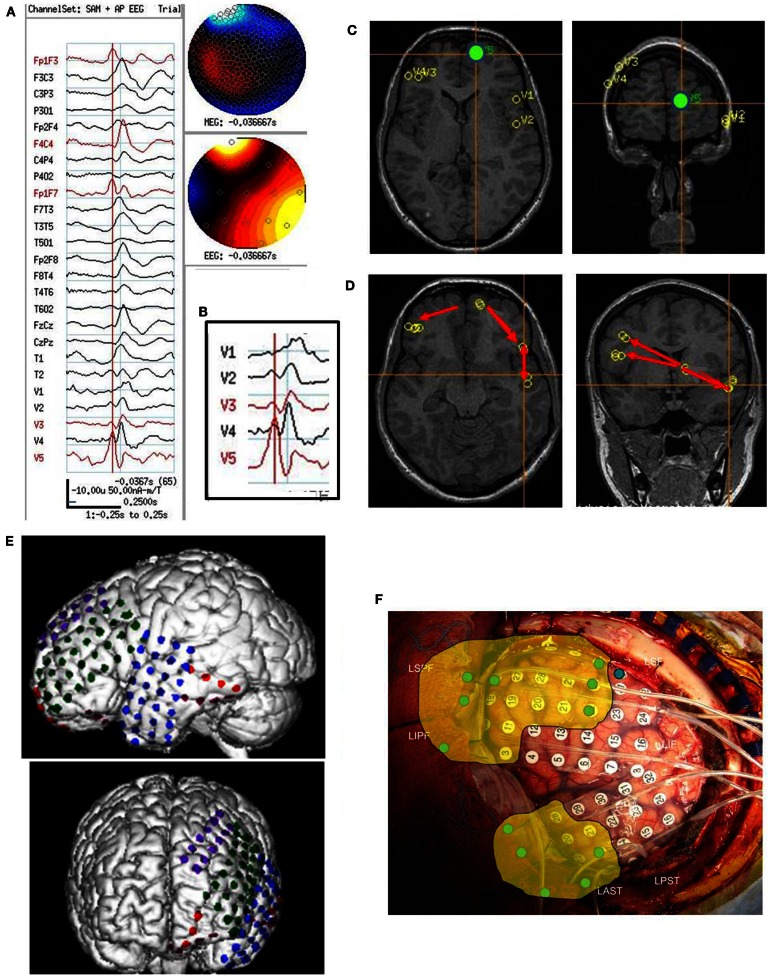
**Subject 1**. **(A)** EEG in bipolar AP montage and five virtual sensor channels showing average of 146 interictal spike epochs in bandwidth 3–70 Hz, digitized at 300 Hz. Topographic maps for MEG and EEG signals are top of head views centered at vertex Cz with anterior (nose) at top of map. For this averaged spike, epochs were aligned based on spike peak detected at VS location V3 in right frontal lobe in the 20–70 Hz bandwidth. **(B)** Inset is enlarged view virtual sensor (VS) channels showing timing of peaks. V5 peak occurs earliest followed by V4 (40 ms), V3 and V2 (50 ms), and V1 (63 ms). Note that although largest amplitude peaks at V1–V4 are delayed after V5 peak, each has a small peak at about the time of V5 peak. Onset of spike peak at V5 location precedes apparent onset at the other four VS locations. Although the averaged epochs shown were aligned based on the spike at V3, the earlier onset of a spike at V5 was detected. **(C)** Subjects MRI scan showing VS locations of V1–V5. Green dot is location of V5, earliest of the VS peaks. **(D)** Subject’s MRI scan showing one possible pattern of spread based on timing of peaks and shortest distances between VS locations. The arrows are only to show relative timing, since propagation and pathways cannot be discerned solely with spike peaks separated in time. **(E)** 3D reconstruction of subject’s MRI and subdural electrode locations. **(F)** Intraoperative photograph of left brain surface. Green dots represent electrodes showing spikes at onset of seizures. Ictal onset was in left frontal lobe at LIPF2–4 (red frontopolar electrodes and purple anterior frontal lobe electrodes) with rapid spread to left anterior temporal lobe (blue electrodes). Yellow overlay represents resection plan.

For each VS location, the peak of the VS spike was used as the time mark to average the spikes at that location. A time window 250 ms before and after the VS spike peak was chosen to make a 500-ms total duration epoch for averaging. The epochs were visually reviewed before averaging, including the simultaneous original EEG and MEG sensor signals. Only epochs that contained just one epileptiform discharge in the EEG/MEG sensor signals were included in the average. The simultaneous EEG, MEG, and VS signals from the other VS locations that occurred during that epoch were averaged at the same time based on the same time mark (Figure 1A). The number of VS spikes that occurred in the VS signals at a VS location varied from 2 to about 200, and in general depended on the sparseness or abundance of epileptiform discharges for that particular subject.

For the purposes of this article, the averaged VS spike at the VS location, along with the simultaneous averaged EEG and MEG sensor signals and the VS signals from the other VS locations, was considered a VS spike “type.” This definition of a “type” is based on a signal occurring at an intracranial location following spatial filtering, although it does include the simultaneous EEG and MEG sensor waveforms and VS waveforms forms from the other VS locations to further characterize the spike “type.” The definition is somewhat different from the conventional definition of an EEG or MEG spike type that is based on the pattern of simultaneous EEG electrode or MEG sensor signals without any spatial filtering.

Since more than one VS location for a subject might have spikes detected in the VS signal, the procedure described above was repeated for the VS spikes at each VS location. Thus, if a subject had five VS locations that had VS spikes, the procedure would be repeated five times. The result would be five sets of averaged VS spikes (five VS spike types) that included the simultaneous EEG/MEG sensor signals and also the simultaneous VS signals from the other locations.

After averaging, the EEG, MEG, and VS averaged spikes were examined for earliest onset of the EEG, MEG, or VS spike and also for time of the waveform peak (Figure 1A). If more than one of the VS locations in an averaged epoch showed VS spikes, the time of VS spike onset and spike peak was compared across each of the VS locations to determine which VS location showed the first spike and the timing difference for EEG, MEG, and VS spikes at the other VS locations (Figure 1A). The averaged epochs from each of the VS locations were compared to determine whether the timing differences for the EEG, MEG, and VS spikes at all VS locations remained consistent.

For subjects showing a consistent timing difference among spikes across VS locations, the unaveraged EEG, MEG, and VS signals were then reviewed again at the time in the original EEG/MEG recording at which the VS spikes occurred to be certain that the timing differences seen in the averages of the EEG/MEG and VS spikes were also present in the unaveraged EEG, MEG, and VS signals.

For the subjects who had interictal or ictal spike bursts in the conventional EEG/MEG signals instead of individual spikes, the simultaneous EEG/MEG sensor signals and VS signals were compared for earliest time of burst onset, but no averaging was done across bursts.

## Results

All subjects had one or more VS locations identified by the SAM + *g*_2_ [SAM(*g*_2_)], evaluation for excess kurtosis. Although the VS locations for each subject had been detected because of excess kurtosis, the subsequent analysis with the adaptive spike detection algorithm did not always detect VS spikes in the VS signals at all the VS locations.

Each VS location/channel that had VS spikes detections that were used to provide the marker for averaging a set of epochs of simultaneous EEG, MEG, and VS signals did show a spike in that location in the bandwidth 20–70 Hz following averaging the epochs, as would be expected. Usually a VS spike was also seen at that VS location in a wider bandwidth filter, such as 3–70 Hz. The other simultaneous VS signals/channels in an averaged epoch also sometimes showed VS spikes.

The VS spikes in the averaged epochs often showed an onset and peak before the simultaneously averaged EEG and MEG sensor spikes. For those averaged epochs that showed VS spikes at multiple VS locations, the timing of onset and peak of the VS spikes by visual inspection appeared near simultaneous across the VS locations, differing by often only 5–20 ms of each other. For a subset of seven subjects the timing of the peaks of VS spikes was longer than 20 ms and more easily discernible by visual inspection.

We studied in greater detail the seven subjects who had the relatively longer time difference among VS spike activation across their VS locations as we felt the slightly greater time separation could be more clearly analyzed. These seven subjects ranged in age from 1 year, 10 months to 16 years, 1 month (mean 9.9 years). Epileptiform patterns in the raw EEG and MEG signals included interictal spikes (three subjects), primarily interictal bursts (two subjects), mixed spikes and bursts (one subject), and both interictal and ictal bursts (one subject). Etiology was probable cortical dysplasia on imaging (one subject), prior brain tumor (one subject), tuberous sclerosis (two subjects), probable encephalitis (one subject) or undetermined at the time of study (two subjects). Of the seven subjects, five had Phase II intracranial monitoring with strip and grid electrode recordings and surgical resection; one subject had corpus callosotomy, and one subject chose not to have any surgical procedure. All six subjects with surgical treatment had a minimum of 2 years follow-up. The seven subjects’ ages, presumed etiologies, epileptiform discharge types and locations, MEG findings, electrocorticography (ECoG) findings, surgical treatments, outcomes, and pathological findings (when available) are summarized in Table 1.

### Subject 1

On video/EEG, the subject had frequent moderate voltage spikes and sharp waves in the left temporal, left frontal, and right frontal head regions. A clinical complex partial seizure was recorded. EEG findings suggested an ictal onset zone at the left frontotemporal region with symptomatic zone at the left temporal head region. On combined MEG/EEG, the subject had 187 spikes and sharp waves that occurred topographically in the left middle temporal head region. SAM(*g*_2_) detected 5 VS locations: left frontal lobe (73 VS spikes averaged), left frontopolar lobe (85 VS spikes averaged), left temporal lobe (107 VS spikes averaged), right frontal lobe (146 VS spikes averaged), right frontopolar lobe (26 VS spikes averaged). The VS location with earliest VS spike was left frontopolar with spread both to right frontal and to left temporal and then to left inferior frontal (Figure 1).

Following craniotomy, subdural electrodes were placed over the lateral cortical surface for left frontal and temporal lobes, left orbitofrontal, and left frontopolar. Ictal ECoG demonstrated two main foci: one in the inferior prefrontal and posterior frontal lobe. In addition, interictal ECoG showed very frequent epileptiform discharges at the left anterior temporal region, which was involved during the two clinical seizures. The subject had a left frontal corticectomy sparing the opercular portion of inferior frontal gyrus and a left anterior temporal lobectomy. At 2 years postoperative follow-up, the subject was seizure free.

### Subject 2

**Figure 2:**
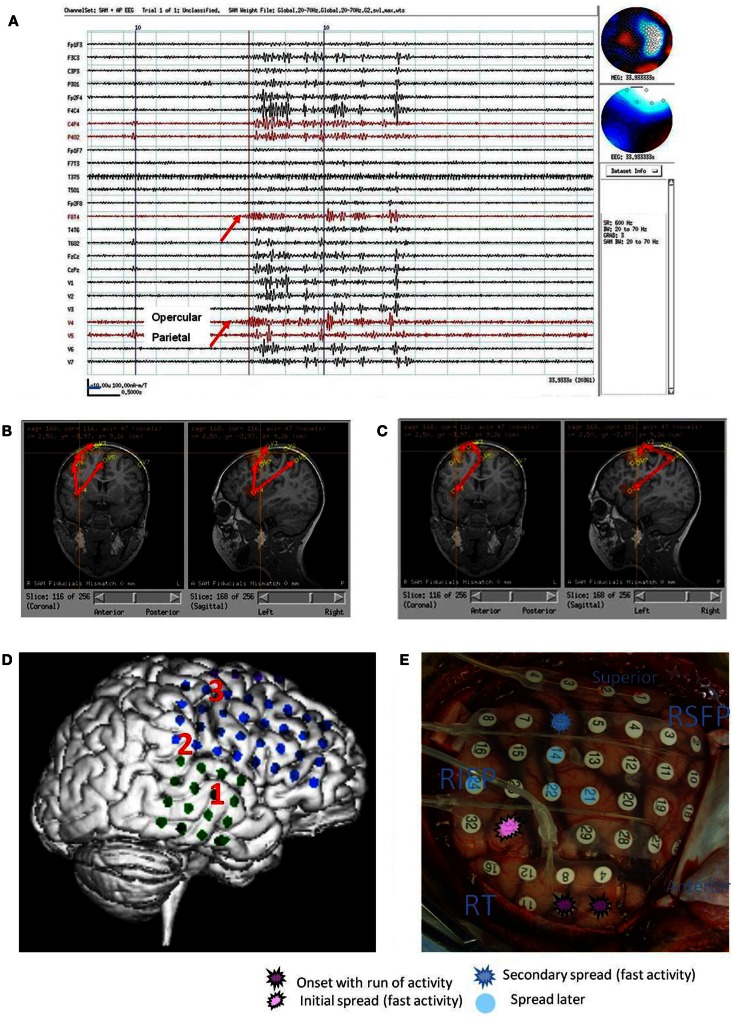
**Subject 2**. **(A)** Bipolar AP EEG and seven virtual sensor channels showing onset of burst in unaveraged signals filtered in bandwidth 20–70 Hz, digitized at 600 Hz. Red arrow at V4, right operculum/insula, shows burst onset preceding first apparent onset in F_8_T_4_ EEG channel by 100 ms. For VS locations, onset in location V4 occurred first followed by right mesial parietal V5 (80 ms), then right mid frontal V7 (240 ms), and right superior mid frontal V3 and V1 (260 ms), and finally right posterior frontal V2 (300 ms). **(B)** Subject’s MRI scan showing one possible pattern of spread based on the timing of the VS peaks. **(C)** Subject’s MRI showing a second timing pattern that occurred in other bursts (not shown) with earliest onset in superior mesial parietal V5, then superior posterior frontal V3 and operculum/insula V4, and then lateral superior midfrontal V1. Red arrows indicate relative timing of peaks. **(D)** Subject’s 3D MRI scan and grid electrode locations. Red numbers 1–3 indicate the ictal onset (1) and spread pattern based on ECoG (2, 3). **(E)** Intraoperative photograph of brain and subdural grids with ictal onset right mid temporal (dark purple), spread to inferior parietal (pink), then superior posterior frontal (dark blue).

On video/EEG frequent spikes occurred in the right hemisphere at O2, P4, and C4. Nine ictal events suggested right parieto-occipital onset. On combined MEG/EEG, the subject showed frequent right parieto-occipital spikes and frequent bursts of polyspikes in the central head regions that were bilateral but more prominent over the right hemisphere. SAM(*g*_2_) analysis of the recording revealed 7 VS locations. Three that showed VS spikes included: right insula (99 VS spikes averaged), right superior posterior frontal (110 VS spikes averaged), and right superior mesial anterior parietal (145 VS spikes averaged). The VS location showing earliest spike activation was right mesial parietal with almost synchronous spread to right posterior frontal and right insula For bursts, the earliest activation was right insula with almost synchronous spread to right posterior frontal and right mesial parietal (Figure 2).

Following craniotomy, subdural electrodes were placed over the lateral cortical surface for right temporal and right frontoparietal with one superior frontal strip. Earliest seizure onset was recorded at the superior temporal electrodes with spread to inferior posterior parietal and later posterior superior frontal. The subject had a right temporal lobectomy and right parietal corticectomy, which on pathology showed diffuse cortical dysplasia. The subject continued to have seizures and required a right hemispherectomy, but has been seizure free at 2 years follow-up.

### Subject 3

**Figure 3:**
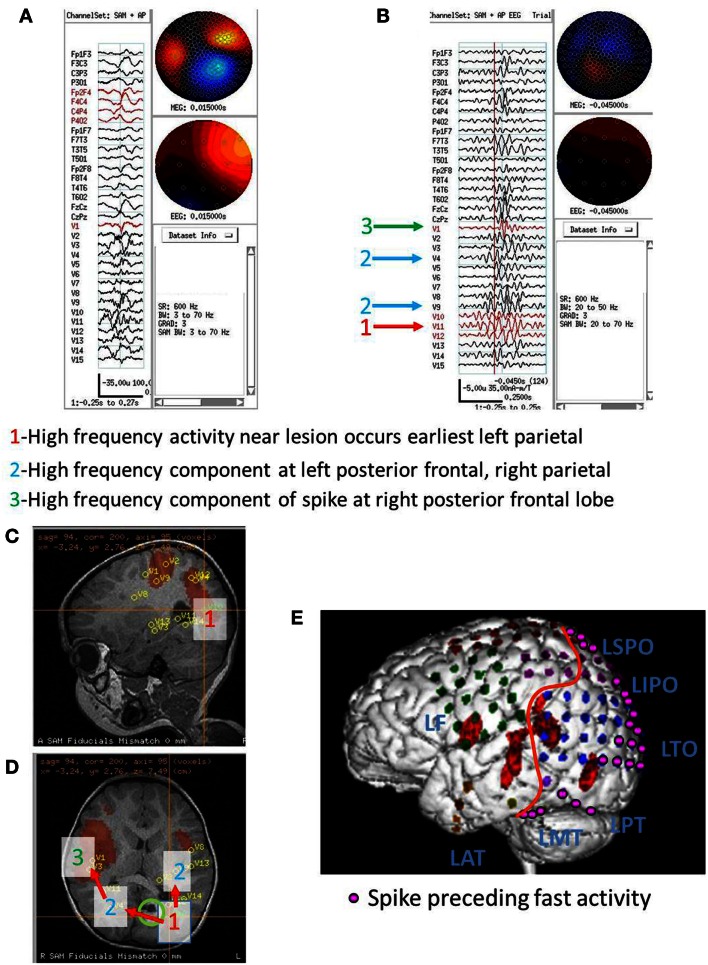
**Subject 3**. **(A)** Bipolar AP EEG and 15 virtual sensor channels showing onset of burst in unaveraged signals filtered in bandwidth 3–70 Hz, digitized at 600 Hz. A spike is seen at V1, but not well seen at other VS locations in this bandwidth (**B)** The same epileptiform discharge but shown in bandwidth 20–70 Hz. Earliest onset at VS location V10 left parietal followed by left posterior frontal V9 (43 ms), and right parietal V4 (43 ms), then right posterior frontal lobe V1 (173 ms). **(C)** Subject’s sagittal MRI scan showing VS location V10 in left parietal at crosshairs and red #1. **(D)** Subject’s axial MRI scan showing location of earliest onset at red #1(V10), then next two onsets at blue #2 (V9 and V4), and then onset at green #3 (V1). Red arrows indicate relative timing of peaks. Green circle indicates location of left parietal MRI lesion. **(E)** 3D reconstruction of subject’s MRI scan. Purple dots indicate the location of spikes on subdural grid electrodes preceding ictal fast activity. Posterior to red line indicates resection plan.

On video/EEG, the subject had frequent multifocal bilateral discharges and multiple clusters of electroclinical seizures that were tonic, atonic, or myoclonic, but electrographically the seizures were similar regardless of semiology. Initial changes appeared to occur in the left posterior region slightly before diffuse changes occurred, suggesting that these could be rapidly generalizing partial onset seizures. MRI imaging had revealed a lesion in the left mesial occipitoparietal region felt to be consistent with cortical dysplasia. On combined MEG/EEG recording, the subject showed multifocal spikes bilaterally and frequent diffuse bursts. SAM(*g*_2_) identified 15 VS locations that showed spike activity during the bursts. These were not averaged, but each burst was individually examined. The earliest onset was in the VS location in the left occipitoparietal junction, just lateral to the MRI lesion. There was spread to VS locations nearby and somewhat later to the left posterior frontal lobe (Figure 3).

Following craniotomy, subdural electrodes were placed over the lateral cortical surfaces for left temporal, parietal, occipital, and posterior frontal lobe. ECoG showed numerous clinical-electrographic seizures arising from the left occipital pole and posterior subtemporo-occipital region spreading both superiorly into the left parietal region and anteriorly into the left temporal region. The surgical resection was left parieto-occipital and posterior temporal. The pathology of the MRI lesion was focal cortical dysplasia. The subject has been seizure free for 2 years.

### Subject 4

**Figure 4:**
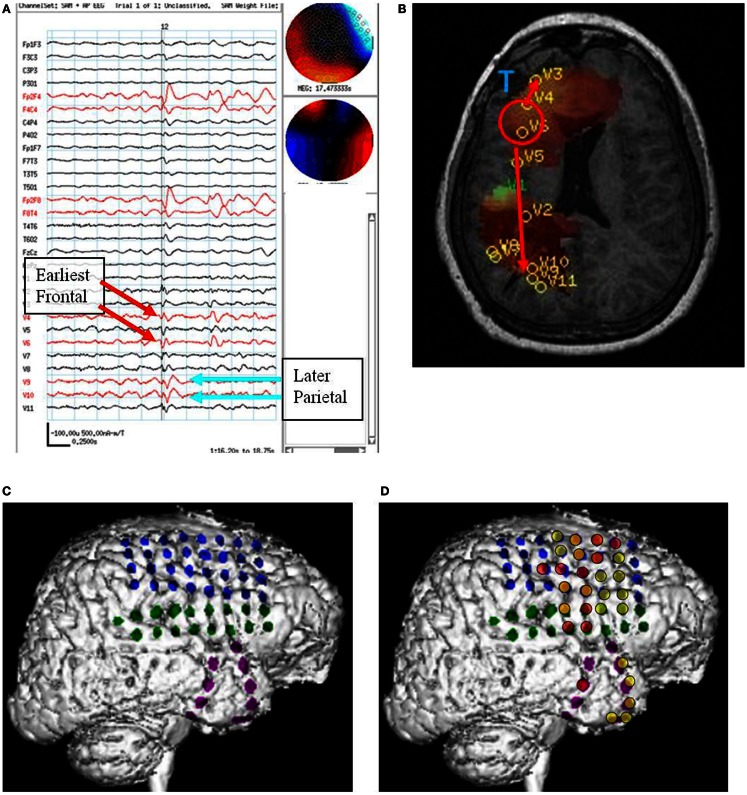
**Subject 4**. **(A)** Bipolar AP EEG and 11 virtual sensor channels showing interictal spike in unaveraged signals in bandwidth 3–70 Hz, digitized at 300 Hz. The earliest peaks occurred at V4 and V6 right frontal lobe (red arrows), just posterior to the resection site for tumor, followed 100 ms later by spike peaks at V9 and V10 right parietal lobe (blue arrows). In an expanded timebased view (not shown), V9 and V10 also show smaller amplitude spikes earlier, 20 ms after V4 and V6. In that view, timing of the spike at V3, right frontal lobe, is also 20 ms after V4, V6. **(B)** Subject’s MRI scan showing tumor resection right frontal lobe (blue T), interictal spike onset at V4, V6 (red circle), and subsequent spike peaks anteriorly at V3 and posteriorly at V9, V10. Red arrows indicate relative timing of peaks. **(C)** 3D reconstruction of subject’s MRI scan with subdural grids over right frontal and temporal lobe. **(D)** Summary of electrodes active at or shortly after seizure onset with the most intense red color corresponding to the most active electrodes in superior, mid, and inferior frontal lobe posterior to resection site and also mid temporal.

This subject had a brain tumor resected in the right frontal lobe 6 years before presurgical epilepsy evaluation and the MEG study. On video/EEG, the subject had frequent high voltage spikes and sharp waves in the left frontal, right anterior temporal, and right frontal head regions. He had 27 events recorded in the EMU, 26 of which were electroclinical seizures: 17 were tonic or generalized tonic clonic seizures (8 localized left frontal head region, 3 to left hemisphere, 6 showed no clear lateralization), 7 were complex partial seizures (5 showed a right frontal localization, 2 did not lateralize), 2 were of atypical semiology and did not show any clear lateralization. On combined MEG/EEG, the subject had 98 spikes seen in the right frontal head region in the EEG recording. The raw MEG signal was obscured by magnetic noise from the subjects vagal nerve stimulator (VNS). SAM(*g*_2_) detected 11 VS locations all in the right hemisphere. Four VS locations were in right frontal lobe (5, 6, 7, and 8 VS spikes), two in right posterior frontal (1 and 7 VS spikes), two in right lateral parietal (4 and 7 VS spikes), and three in right mesial parietal (1 VS spike in each VS location). The spikes in the right frontal lobe preceded those in the right mesial parietal lobe by about 65 ms (Figure 4).

Following craniotomy, subdural electrodes were placed over the lateral cortical surfaces for right frontal and anterior parietal lobe, and strips over lateral and inferior temporal lobe. ECoG recorded five clinical seizures characterized by asymmetric tonic posturing and one myoclonic seizure. The seizure onset was localized to the right mid and anterior frontal region with rapid spread to right anterior and mid temporal regions. A right frontal resection was performed. Postoperatively the subject had recurrent seizures.

### Subject 5

**Figure 5:**
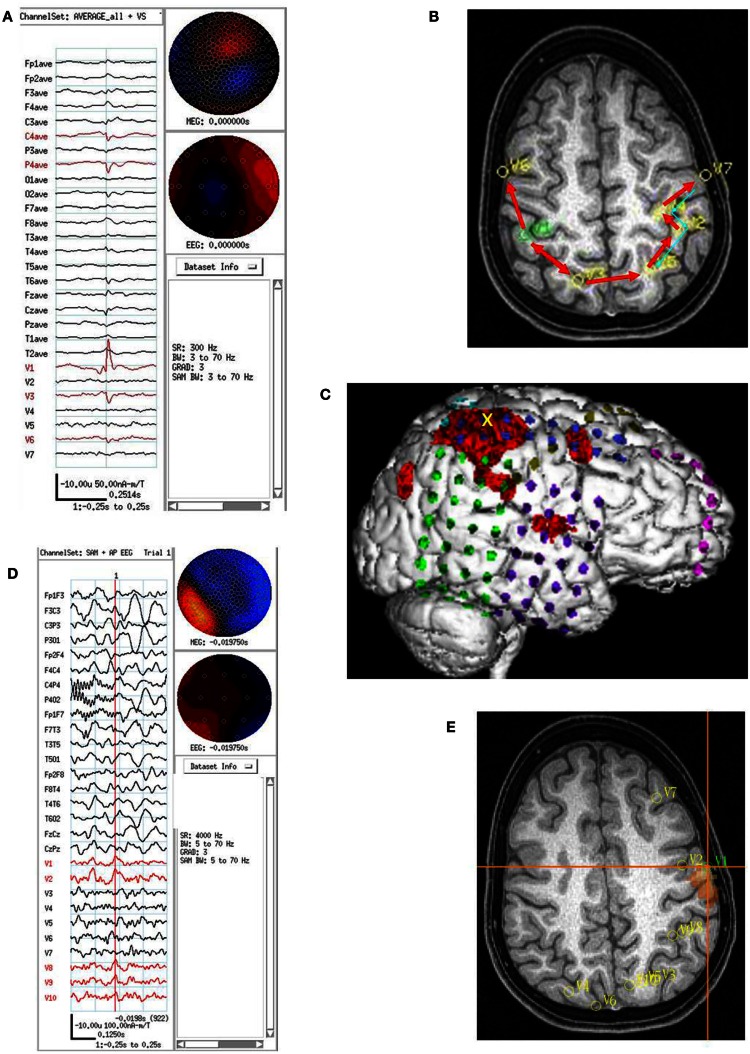
**Subject 5**. **(A)** EEG in average reference montage and seven virtual sensor channels showing average of 72 interictal spike epochs in bandwidth 3–70 Hz. For this averaged spike, epochs were aligned based on spike peak detected at VS location 1 in right parietal lobe in the 20–70 Hz bandwidth. The earliest onset occurred in V1 about 33 ms before the first onset in EEG channels C4 and P4. The panel also shows the first peak in V1 preceded the peak in V3 right mesial parietal and V6, right posterior frontal. Averages of epochs based on the timing of the spikes at each of the other six VS locations (not shown) were compared to obtain a composite of spike peak timing differences among all VS locations. **(B)** Subject’s MRI scan showing the composite of spike peak timing differences. Red arrows indicate relative timing of peaks. The average of epochs based on MEG spike detections in the 20–70 Hz bandwidth for VS location V7 showed an additional pattern of spike peak timing that tracked back from left posterior frontal to anterior parietal, then posterior parietal, but did not cross the midline to the right hemisphere. **(C)** 3D reconstruction of the subject’s MRI scan showing subdural electrode locations over right frontal, parietal, and temporal lobes. Ictal onset was in superior anterior parietal at the yellow X. [The red regions here represent region of activation from subtraction ictal SPECT co-registered with MRI (SISCOM) but were not analyzed as part of the current study.] **(D)** Combined EEG/MEG study performed 2 months postoperatively because subject continued to have seizures. Bipolar AP EEG and 10 virtual sensor channels showing average of 3 interictal spike epochs in bandwidth 5–70 Hz, digitized at 4 KHz. The peaks at VS locations 1–2 and 8–10 all seem to occur at approximately the same time. Spikes were seen in the MEG signals, but not well seen in the EEG. **(E)** The subject’s MRI scan showing the VS locations from the postoperative MEG. Highest excess kurtosis was at left posterior frontal near V1 and V2 (red region). This location was also near the location of the second spread pattern seen preoperatively (Panel B) in left posterior frontal (V7 location in that panel).

On video/EEG, the subject had occasional spike and slow waves in the left frontocentral and left centroparietal head regions. Twelve tonic seizures were recorded: nine began in the right temporal head region, one in the right centroparietal region, one in the left centroparietal region, and one was poorly localized. On combined MEG/EEG, the subject had multifocal interictal spikes: left frontal, left frontocentral, left centroparietal, right centroparietal, and right frontocentral head regions. SAM(*g*_2_) detected multiple VS locations bilaterally with VS spikes in left lateral superior frontal lobe (21 VS spikes averaged), left lateral superior parietal lobe (anterior – 5 and posterior – 17 VS spikes averaged), right frontal lobe (10 VS spikes averaged), right parietal lobe (lateral anterior superior – 72 and lateral mid superior – 20 VS spikes averaged). Based on the order of occurrence of VS spikes in the right hemisphere, the anterior and posterior parietal lobe locations showed the earliest spike, with spread into the right posterior parietal lobe and right lateral midfrontal lobe VS spike. In addition, the order of occurrence of VS spikes suggested there was simultaneous spread from the right posterior parietal to left posterior parietal and then to left anterior parietal, left posterior frontal, and left lateral midfrontal lobe locations. A second pattern of spread (again based on VS spike order of occurrence, but also based on averaging left hemisphere interictal spikes) suggested that some VS spikes began in the left lateral midfrontal lobe and spread to left posterior frontal lobe, then to left anterior parietal lobe locations. This left lateral midfrontal lobe VS location was both the last VS location showing spread from the right parietal spikes and the first VS location showing activation and spread of VS spikes for spikes originating in the left hemisphere (Figure 5).

Based on the multimodality presurgical studies in addition to MEG, a right craniotomy was performed with electrode grids placed over the right frontal, temporal, and parietal lobes. Five typical seizures were recorded with onset detected in the superior right frontal lobe on the right mid superior frontoparietal grid, frontal grid, and middle interhemispheric strip. A right posterior frontal lobe resection was performed with multiple subpial resections over the motor cortex. Initially postoperatively the subject was seizure free. However, within 1 week seizures recurred. On repeat video/EEG, the subject showed a different clinical pattern of flexing the right elbow and flexing the back with EEG onset in the left central head region. Combined MEG/EEG was repeated at that time. The subject had fewer epileptiform discharges per unit time compared to the first study. The most frequent detections of VS spikes were in the left lateral midfrontal lobe location described above that preoperatively showed late activation after right parietal VS spikes and earliest activation of VS spikes for left hemisphere epileptiform discharges. The family declined a second operative procedure, and a second Phase II study was therefore not indicated. At 2 years postoperative follow-up, the subject continued to have intermittent seizures.

### Subject 6

**Figure 6:**
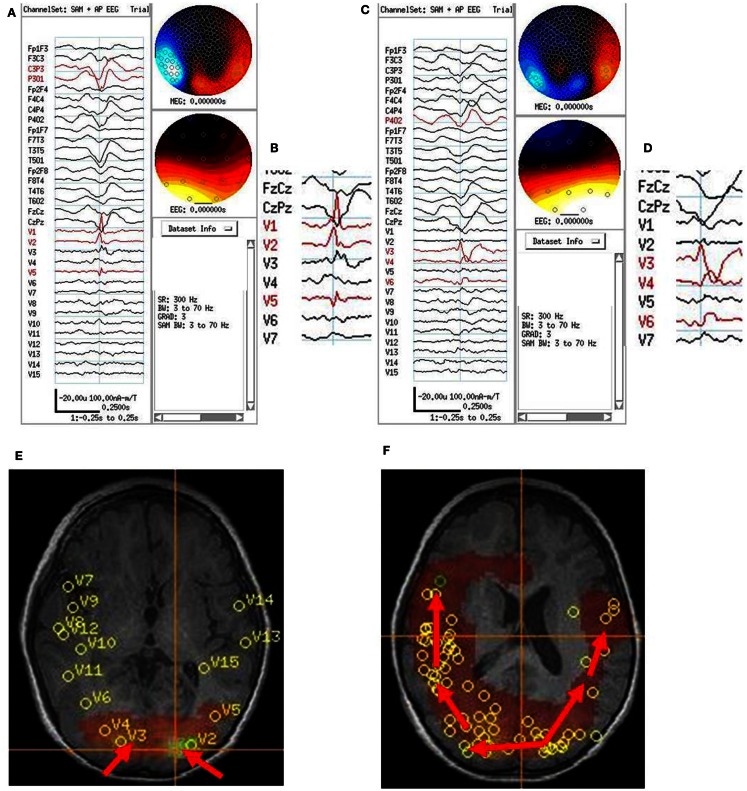
**Subject 6**. **(A)** Bipolar AP EEG and fifteen virtual sensor channels showing average of 154 interictal spike epochs in bandwidth 3–70 Hz. For this averaged spike, epochs were aligned based on spike peak detected at VS location V1 in left occipital lobe in the 20–70 Hz bandwidth. **(B)** Inset shows close-up of VS peaks at VS locations. Although the averaged epochs shown were aligned on the timing for VS location V1, the average shows the earliest VS spike onsets were at VS locations V2 and V5 left occipitotemporal, then 13 ms later spike peak at V1, left occipital, and 13–26 ms later two peaks in V3, right occipital. **(C)** Bipolar AP EEG and fifteen virtual sensor channels showing average of 129 interictal spike epochs in bandwidth 3–70 Hz. For this averaged spike, epochs were aligned based on spike peak detected at VS location V3 in right occipital lobe in the 20–70 Hz bandwidth. **(D)** Inset shows close-up of VS peaks at VS locations. The spike peak at V3, right posterior occipital occurred earliest followed by V4 (26 ms), right anterior occipital, and V6 (39 ms), right posterior temporal. **(E)** Subject’s axial MRI scan showing right V3 and left V2 occipital earliest spike peak locations. **(F)** Subject’s 3D MRI scan showing relative timing of peak locations starting in either left or right occipital lobe and spreading anteriorly. Red arrows indicate relative timing of peaks, pointing toward later peaks.

On video/EEG, the subject had frequent myoclonic and atonic seizures with diffuse electrographic changes that were not well-localized. On combined MEG/EEG, the subject had very frequent bilaterally diffuse and synchronous spikes and polyspikes. SAM(*g*_2_) detected multiple VS locations in the parietal and occipital lobes and fewer in the temporal and posterior frontal lobes bilaterally. Two VS locations that showed prominent VS spikes were left (154 VS spikes averaged) and right (129 VS spikes averaged) posterior parietal lobes. The left parietal VS spike usually preceded the right parietal VS spike by about 25 ms (Figure 6). Based on all of the subject’s tests, the subject was judged clinically not to be a candidate for focal resection, did not have a Phase II study, but did have a corpus callosotomy. At 2 years follow-up, the subject continued to have seizures, but not the drop attacks.

### Subject 7

**Figure 7:**
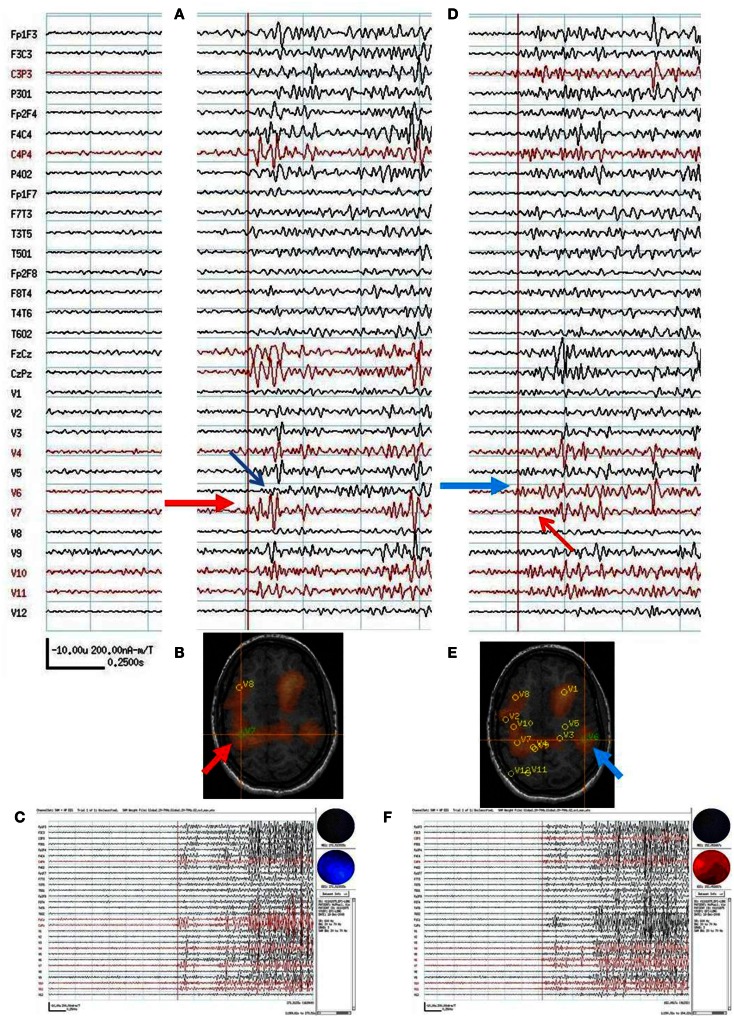
**Subject 7**. **(A)** Bipolar AP EEG and 12 virtual sensor channels showing onset of generalized ictal onset in unaveraged signals filtered in bandwidth 20–70 Hz, digitized at 600 Hz. Red arrow indicates VS location V7 in right parietal. Ictal onset at V7 precedes by 120 ms the ictal onset in left parietal V6 (blue arrow). **(B)** Subject’s MRI scan showing VS V7 location in right parietal lobe (at red arrow and crosshairs). **(C)** Overview of ictal onset from right parietal lobe. **(D)** Second generalized ictal onset. Blue arrow indicates VS location V6 in left parietal Ictal onset at V6 precedes by 83 ms the ictal onset in right parietal V7 (red arrow). **(E)** Subject’s MRI scan showing VS V6 location in left parietal lobe (at blue arrow and crosshairs). **(F)** Overview of ictal onset from left parietal lobe.

On video/EEG, the subject had frequent spikes and sharp waves in the left anterior temporal region head region. Frequent bursts of high voltage diffuse spikes and polyspikes and slow waves occurred with a frontocentral predominance. The subject had brief tonic seizures lasting 3–5 s and multiple complex partial seizures, but with somewhat variable semiology. All clinical seizures were poorly localized or lateralized on scalp EEG. On combined MEG/EEG, the subject had frequent focal spikes bilaterally and multiple bursts of polyspikes seen on EEG, six of which were associated with a bilateral tonic “shudder” of the arms. The MEG sensor signals could not be directly interpreted because of the magnetic noise from the subject’s VNS. The spatial filter characteristics of the beamformer algorithm excluded this distant noise source in the SAM VS. SAM(*g*_2_) detected VS locations in left frontal lobe (424 VS spikes averaged), right frontal lobe (472 VS spikes averaged), and right opercular (106 VS spikes averaged), right parietal (118 VS spikes averaged), and occipital lobes (122 VS spikes averaged). No consistent pattern of spread from one VS location to another (based on VS spike onset or peaks) was found for averaged VS spikes. For the six brief clinical seizures, sometimes the earliest VS spikes (in the VS signals associated with the EEG polyspike bursts) was in the right hemisphere and at other times in the left hemisphere in the 20–70 Hz bandwidth (Figure 7). The VS waveforms at locations of peak spikiness suggested that the location of earliest onset during the electroclinical seizures of polyspike bursts varied and could begin in either the right or left hemisphere. The subject chose not to proceed with surgery, and Phase II evaluation was therefore not done.

## Patterns of VS Spike Occurrence during EEG/MEG Sensor Interictal and Ictal Discharges

The spatial separation between VS locations showed that spread of activation varied from intralobar (five subjects) through intrahemispheric (seven subjects) to interhemispheric (five subjects), with six subjects showing more than one distance of spread. Timing for spread intralobar ranged from 20 to 63 ms, intrahemispheric was 39 to 300 ms, and interhemispheric was 26 to 173 ms (Table 1; Figures 1–7). Usually, the VS location that showed the earliest visually noticeable change from baseline also showed the earliest peak activity; however, sometimes two VS locations showed approximately similar first change from baseline, but one of the two VS locations might demonstrate an earlier peak. For some subjects, the VS locations that showed delayed spike peaks sometimes also showed a small amplitude deflection shortly after the VS spike at the VS location showing earliest VS spike peak.

## Comparison of Preoperative MEG Beamformer Putative Source Timing and Intracranial ECoG Interictal/Ictal Recordings

For comparison of preoperative MEG beamformer predictions and ECoG findings, two of the seven subjects did not proceed to Phase II evaluation with ECoG, so for those two subjects no comparison between MEG and ECoG can be made.

For subject 1, MEG beamformer showed left frontopolar onset with spread to left temporal lobe, both of which were detected on ECoG and in that order for ictal onsets. However, later spread to the left inferior frontal was not detected, which could be MEG beamformer error, or possibly the left frontal grid did not extend sufficiently inferiorly over frontal lobe. ECoG could neither validate nor invalidate occurrence or relative timing of VS spikes in the right frontal lobe, since no intracranial electrodes were placed over that hemisphere. The subject has been seizure free for 2 years without any resection in right frontal lobe.

For subject 2, MEG beamformer detected an onset in right temporofrontal operculum or insula and subsequent occurrence in right mesial parietal, right mid frontal, and superior frontal. ECoG, on the surface of the frontal and temporal lobes, detected an onset in right middle temporal gyrus, then inferior parietal, and finally anterior superior parietal or posterior superior frontal. Overall both preoperative MEG beamformer and ECoG showed origin near/in temporal lobe with subsequent spread dorsally to parietal and then superior parietofrontal. No intracranial recording, stereotaxic/stereotactic (SEEG), was done of the insula/opercula nor ECoG of superior mesial parietal, so intracranial recordings did not validate or invalidate either location as an onset site or terminal extension of ictal activity as suggested by MEG interictal VS fast activity burst analysis. The resection was limited to temporal and inferior parietal, but the subject continued postoperatively to have seizures. Subsequently the subject had right hemispherectomy and thereafter has been seizure free for 2 years.

For subject 3, MEG beamformer showed left parietal onset in sulci just lateral to the MRI lesion followed both left posterior frontal and right parietal to right posterior frontal spread. ECoG-detected spikes in the left posterior parietal and occipital surfaces and rapid diffuse spread into the left anterior parietal and posterior temporal lobes, but not into left posterior frontal lobe. No intracranial electrodes were placed over the right hemisphere, so spread and timing there could not be validated/invalidate by the intracranial recordings. The resection was limited to left occipitotemporoparietal lobes, but the subject was 2 years seizure free.

For subject 4, MEG beamformer showed a frontal onset posterior the prior resection site and both more anterior spread mesially in frontal lobe and posterior spread to deep in the parietal lobe. ECoG showed ictal onset posterior to the lesion in frontal lobe but did not detect the parietal spread shown in MEG and frontopolar surface intracranial electrodes were not placed, perhaps because of scarring from the prior tumor resection. After a prefrontal lobe resection, 4 cm from the frontal tip, the subject continued to have seizures.

For subject 5, MEG beamformer detected a right frontoparietal onset but also a separate left posterior frontal onset. The other modality tests done clinically preoperatively also pointed to right frontoparietal region. ECoG over right temporofrontoparietal regions showed superior right parietal onset. No ECoG was done over the left hemisphere, so a second left posterior frontal onset could not be validated/invalidated. After a right posterior frontal lobe resection with multiple subpial resections over the motor cortex, the first ictal clinical pattern disappeared, but new seizures recurred suggesting left hemisphere onset based on semiology. A postoperative MEG showed left posterior frontal and parietal VS spikes that were not well seen in simultaneous 10–20 scalp EEG. No further intracranial EEG studies were planned as a second resection was not planned, so the persistent left posterior frontal MEG spikes could not be confirmed as the ictal onset location in the left hemisphere.

## Discussion

This study examined source localization for epileptiform discharges with a beamformer algorithm that spatially filtered the MEG signals by “tuning” the MEG sensor array to provide a single VS signal at a single VS location. Each 2 or 10 min MEG recording was iteratively examined offline at multiple intracranial locations for source activity. The source activity at each location was evaluated with the *g*_2_ excess kurtosis statistic.

Virtual sensor signals at the VS locations with highest excess kurtosis were evaluated for transient spikes. When spikes were detected, epochs surrounding the spikes were averaged including the simultaneous EEG/MEG sensor spikes and simultaneous VS signals at up to the 20 VS sensor locations with the highest excess kurtosis.

In review of all 162 subjects, the MEG VS spikes often had their onset at, or sometimes just before, the onset of the EEG/MEG sensor spikes (see Figures 2 and 5) and sometimes were shorter in duration than the associated EEG/MEG sensor spikes (see Figure 6). The finding of an earlier VS onset may have occurred because the spatial filtering lowered the contribution of background noise from other sites in the brain, improved the signal to noise ratio, and allowed a smaller amplitude, earlier occurring spike signal to be detected above the lowered background noise level. The shorter duration spike may indicate that activity at that location had ceased and propagated to another site; alternatively, the finding may indicate local contiguous spread created a more extended source that fit less well the forward solution of a focal dipole source, and lead to a lower amplitude VS signal.

For most of our 162 subjects whose averaged epochs contained VS spikes at multiple VS locations, and the timing of onset and peak of the VS spikes across the VS locations was similar. Based on visual inspection, these VS spike features appeared to occur either synchronously or varied by just 5–20 ms in timing. For a subset of seven subjects the timing of VS spike onsets and peaks differed more, and differences were discernible with visual inspection. Although time for a signal to cross from one cerebral hemisphere to the other has been quoted as 10 ms (Barth et al., 1982) for axonal transmission through the corpus callosum, the timing differences between component VS spikes of the EEG/MEG sensor spike were greater than 10 ms. The reason for the greater delay seen for our seven subjects is not certain. The spikes in the original simultaneous scalp EEG did not appear unusual, although the epileptiform discharge for subject 6 was actually a sharp wave by time duration. For these subjects the delay may relate to a time period required for one cortical region to activate another cortical region. Similar delays in propagation time of interictal spikes were noted previously in a subset of subjects in a combined scalp and intracranial EEG study; however, in those subjects also nothing strikingly unusual was noted in the morphology of the scalp EEG spikes and/or sharps waves (Alarcon et al., 1994). As noted in the results section, for some subjects, the VS locations that showed delayed spike peaks sometimes also showed a small amplitude deflection shortly after the VS spike at the VS location showing the earliest VS spike peak. Axonal transmission times might be appropriate from one intralobar, intrahemispheric, or interhemispheric location to another, but activation of independent synchronized activity at the secondary sites might require the longer time delays.

## Comparison of Preoperative MEG Beamformer Putative Source Timing and Intracranial ECoG Interictal/Ictal Recordings

Although two of seven subjects did not proceed to Phase II intracranial recordings, the MEG beamformer and intracranial EEG (ECoG) did share similarities of at least onset locations for the remaining five subjects. Corroboration of patterns of spread, predicted by MEG, was seen to a certain extent in for subjects 1 and 2 in the ECoG.

Several differences of the two recording modalities became relevant for attempts to corroborate non-invasive MEG prediction of onset locations and spread patterns using intracranial EEG recordings. First is the physics limitation that MEG is more likely to record tangential sources in sulci, which ECoG may not detect well, while ECoG records radial sources on the surface of the brain, the gyral crowns, which MEG does not detect well (Hillebrand and Barnes, 2002). Thus some spread to deeper locations may not be detected by ECoG, while spread from one gyral crown to another, perhaps by *U*-fibers, may not be detected by MEG (e.g., Figure 2, subject 2 – the second ECoG-detected location in lateral inferior parietal cortex but not detected by MEG).

The limitations of ECoG to see deeper sources in sulci may in part be solved by combined SEEG and ECoG, or just SEEG, but considerable preoperative planning is required for either evaluation (Koessler et al., 2010; Kakisaka et al., 2012). Non-invasive preoperative MEG detection of multiple contributory sources relative to gyral crown components may improve with simultaneous higher resolution scalp EEG with more closely spaced electrode placement such as 64, 128, or 256 channel arrays (Yamazaki et al., 2012). Beamformer analyses of EEG scalp recordings have been reported, but success may be dependent on the accuracy of the forward model and knowledge of variations in skull impedance over the head (Steinstrater et al., 2010; Dang et al., 2011; Jon Mohamadi et al., 2012). Combined beamformer analysis with MEG and EEG may provide improved timing details regarding activation of cortical sources with both radial and tangential current sources (Schoffelen and Gross, 2009; Shahbazi Avarvand et al., 2012).

Postoperative outcomes may suggest support for sources seen in MEG beamformer, but not detected with intracranial electrodes, when those MEG identified structures have not been resected. This difference may be the case for the insular and mesial parietal MEG sources for subject two that were not resected initially and seizures continued, but were resected with the subsequent hemispherectomy and seizures ceased. The difference could also be the case for a deep parietal source for subject four predicted by MEG that was not resected and seizures continued in that subject. Nonetheless, the persistence of seizures after MEG-predicted source locations that were not resected, or the disappearance of seizures after procedures like hemispherectomy, does not validate or invalidate precise location predictions of MEG.

Another limitation occurs when MEG detects bilateral sources, but subsequent invasive intracranial EEG is performed only for one hemisphere. The features that make MEG detection of interhemispheric spread clinically relevant are not yet delineated. For subjects 1 and 3, interhemispheric spread of interictal spikes without resection of the MEG-detected sources in the contralateral hemisphere were not relevant, as without their resection, the subjects still had a seizure free outcome 2 years postoperatively. By contrast, the detection of spread of interictal spikes to a site in the contralateral hemisphere in subject five, and detection of independent spikes that arose from that contralateral site and spread locally there, did presage the subject’s postoperative seizures in that contralateral hemisphere. Numbers of intracranial electrodes that can be placed clinically safely are limited, so extensive bihemispheric ECoG or SEEG coverage will not likely be the solution. Better understanding of the clinical relevance of spread of spikes to a contralateral hemisphere may require comparing MEG studies with other non-invasive whole-head modalities such as the high density scalp EEG recordings or EEG-fMRI in addition to tracking surgical outcomes.

## Macro Networks in Clinical Epilepsy

The concept of a single epileptic focus to be surgically removed was popular in the early years of epilepsy surgery. The concept may be very appropriate still for seizures limited to mesial temporal lobe. However, in pediatrics most medically intractable epilepsy is extratemporal in origin and may originate in more than one location, especially when cortical dysplasia is involved.

Networks in the propagation of seizures can occur at multiple levels (Lemieux et al., 2011). At a single cortical location, a local network over a few square centimeters of cortex may be required to sustain repetitive fast activity at the beginning of a seizure. Larger networks may involve a single or several contiguous gyri and may include both a SOZ and a surrounding region of rapid spread to give an EZ, which must be resected entirely to achieve seizure control (Engel, 1996; Wiebe et al., 2001; Luders et al., 2006). A still larger network may involve a SOZ with a region of rapid spread that may be located several gyri away or even in a different lobe or the contralateral hemisphere. For these larger networks there may be more than one cortical region with lower threshold for seizure onset and for ictal spread. Based on clinical experience with ECoG, when more than one cortical region is present with apparent low ictal threshold, the roles of SOZ and region of rapid spread may alternate between the two or more cortical regions, depending on the particular ictal event evolving at the time. For these latter cases, surgical treatment may involve resections or corticectomies at multiple sites, or sometimes the decision not to proceed with resective surgery.

The hope and expectation of clinicians treating pediatric medication resistant epilepsy is that intracranial electrodes can be placed during invasive monitoring to identify and delineate all the SOZs and all regions of rapid spread. Preoperative identification of multiple intracranial sources contributing to the interictal epileptiform discharge or ictal event is an important goal. The knowledge may help the neurosurgeon better plan the surgical approach to place intracranial electrodes (Knowlton et al., 2009).

Accomplishing this goal based on scalp EEG may be difficult using the conventional 10–20 electrode set and head surface topography of scalp voltage potentials. Efficacy may be improved with additional scalp electrodes, though multiple intracranial sources may be difficult to delineate with scalp recording topography, since EEG sees a composite of radial and tangential dipoles; source localization algorithms may be required (Ebersole, 1994; Ochi et al., 2001).

MEG studies usually require mathematical algorithms to predict intracranial source locations. The conventional algorithm is the single ECD, but in its simplest form it localizes a single source. When two or more sources are active simultaneously or the timing of their activation overlaps, the single ECD may predict a location between the two sources, which in some cases may mislocalize to white matter or ventricle. The moving ECD algorithm can identify that the average location of the composite of sources changes over time, but the algorithm is not configured to identify where individual contributing sources are located. Current source density algorithms may better account for multiple sources; however, in general the timing of activation of the different source may be somewhat difficult to ascertain based on the current density map (de Gooijer-van de Groep et al., 2012). All of the algorithms that do not use spatial filtering suffer from including fluctuations in background noise in the source localization calculations. Although keeping the subject quiet, relaxed, and still are important for reducing extracranial noise sources, intracranial brain noise fluctuations are ever present and are still part of the signal that is being localized (Ward et al., 1999).

On the other hand, every methodology has limitations. For clinical studies we also evaluate the traditional ECD model and both dipole scan algorithms (MUSIC) and current density algorithms (MNE, sLORETA). A limitation for beamformers is that two sources that changed together with identical relative amplitude time courses, but at different locations, could not be distinguished by a beamformer algorithm and would be mislocalized to a third location (Diwakar et al., 2011). The observation that beamformers do as well as they do, suggests that different brain regions most often do not have exactly identical relative amplitude time courses, particularly at the higher frequency bandwidths and if greater than 3 cm distant from one another (Belardinelli et al., 2012; Moiseev and Herdman, 2013).

In this study we first visually inspected the EEG/MEG signals for artifact, then used quantitative algorithms to (1) spatially filter the MEG signal, (2) measure excess kurtosis to identify candidate brain regions of spikiness, and (3) identify timing of the transient spikes in the regions that contributed to the spikiness of the signal. Then we returned to simple visual inspection of the VS signals to compare the timing of the original EEG/MEG interictal and ictal discharges with the VS spike timing at each of the VS locations. We found that multiple sources identified by this method were seen also in the ECoG in similar, but not exactly identical, cortical locations. At this time it is not certain whether the differences in locations between ECoG and beamformer were secondary to beamformer relative mislocalization, or whether the different cortical substrates measured (ECoG – gyral crowns; beamformer – gyral sulci), contributed to some of the differences.

Visual inspection alone was able to detect timing differences among the EEG/MEG spikes and the VS signals in 7 of 162 (4%) subjects, which is a low percentage of all subjects. However, visual inspection of signals should be done at each step of analysis/processing of the signals to obtain a Gestalt of what changes are occurring in the signals and for quality control to detect signal artifacts in what may become clinical tools. The range of differences in timing VS spikes for the remaining 155 subjects were in the range of 5–20 ms. The signal digitization rates we are using now (600–4 KHz) may allow us to use pursue quantitative comparisons after visual inspection, to evaluate better the timing differences in onset and peak across VS spikes and EEG/MEG sensor spikes.

There are several limitations to the current study. The sample size is a small percentage of all subjects we studied in the enrollment time window, as noted above. Only five of seven subjects had resective surgery, and for subject 5, a second ECoG recording was not done after the second MEG study. Only three of the five with resective surgery were seizure free; a fourth (subject 5) who did not have the contralateral source resected, continued to have seizures. Although that finding might be considered supportive, subject 5 did not have a second ECoG, so the contralateral source could not be confirmed as a second SOZ. For these reasons, the clinical significance of VS spike activation delay remains uncertain until a larger population can be examined and the degree of usefulness of the analysis can be better understood.

## Conclusion

A spatial filter analysis of MEG signals such as a beamformer may be helpful during presurgical epilepsy evaluation to distinguish subregions of spiking during recorded interictal spikes and onsets of ictal discharges. In a subset of subjects the differences in timing between these onsets may be great enough to be apparent by visual inspection. For our subject subset the onset and timing differences roughly correlated with the SOZs and regions of spread determined by later ECoG. These subjects appeared to have a network of spread of the seizures within a cerebral lobe, across lobes and from one hemisphere to another. If the significance of VS spike activation delay can be better ascertained in a larger population, this kind of presurgical evaluation may be helpful for presurgical planning for surgical approach and intracranial electrode placement.

## Conflict of Interest Statement

The authors declare that the research was conducted in the absence of any commercial or financial relationships that could be construed as a potential conflict of interest.
